# Sphingolipid-mediated inflammatory signaling leading to autophagy inhibition converts erythropoiesis to myelopoiesis in human hematopoietic stem/progenitor cells

**DOI:** 10.1038/s41418-018-0245-x

**Published:** 2018-12-13

**Authors:** Marion Orsini, Sébastien Chateauvieux, Jiyun Rhim, Anthoula Gaigneaux, David Cheillan, Christo Christov, Mario Dicato, Franck Morceau, Marc Diederich

**Affiliations:** 1 Laboratoire de Biologie Moléculaire et Cellulaire du Cancer, Hôpital Kirchberg, 9, rue Edward Steichen, 2540 Luxembourg, Luxembourg; 2 Department of Pharmacy, Research Institute of Pharmaceutical Sciences, College of Pharmacy, Seoul National University, 1 Gwanak-ro, Gwanak-gu, Seoul 08826 South Korea; 3 Service de Biochimie et Biologie Moléculaire Grand Est, Unité Médicale Pathologies Métaboliques Erythrocytaires et Dépistage Périnatal, Centre de Biologie et de Pathologie Est, Hospices Civils de Lyon, Bron, France; 4 Service Commun de Microscopie, Université de Lorraine, Nancy, France

**Keywords:** Experimental models of disease, Chronic inflammation, Macroautophagy, Haematological diseases

## Abstract

Elevated levels of the pro-inflammatory cytokine tumor necrosis factor-α (TNFα) inhibit erythropoiesis and cause anemia in patients with cancer and chronic inflammatory diseases. TNFα is also a potent activator of the sphingomyelinase (SMase)/ceramide pathway leading to ceramide synthesis and regulating cell differentiation, proliferation, apoptosis, senescence, and autophagy. Here we evaluated the implication of the TNFα/SMase/ceramide pathway on inhibition of erythropoiesis in human CD34^+^ hematopoietic stem/progenitor cells (CD34/HSPCs) from healthy donors. Exogenous synthetic C2- and C6-ceramide as well as bacterial SMase inhibited erythroid differentiation in erythropoietin-induced (Epo)CD34/HSPCs shown by the analysis of various erythroid markers. The neutral SMase inhibitor GW4869 as well as the genetic inhibition of nSMase with small interfering RNA (siRNA) against sphingomyelin phosphodiesterase 3 (SMPD3) prevented the inhibition by TNFα, but not the acid SMase inhibitor desipramine. Moreover, sphingosine-1-phosphate (S1P), a ceramide metabolite, restored erythroid differentiation, whereas TNFα inhibited sphingosine kinase-1, required for S1P synthesis. Analysis of cell morphology and colony formation demonstrated that erythropoiesis impairment was concomitant with a granulomonocytic differentiation in TNFα- and ceramide-treated EpoCD34/HSPCs. Inhibition of erythropoiesis and induction of granulomonocytic differentiation were correlated to modulation of hematopoietic transcription factors (TFs) GATA-1, GATA-2, and PU.1. Moreover, the expression of microRNAs (miR)-144/451, miR-146a, miR-155, and miR-223 was also modulated by TNFα and ceramide treatments, in line with cellular observations. Autophagy plays an essential role during erythropoiesis and our results demonstrate that the TNFα/neutral SMase/ceramide pathway inhibits autophagy in EpoCD34/HSPCs. TNFα- and ceramide-induced phosphorylation of mTOR^S2448^ and ULK1^S758^, inhibited Atg13^S355^ phosphorylation, and blocked autophagosome formation as shown by transmission electron microscopy and GFP-LC3 *punctae* formation. Moreover, rapamycin prevented the inhibitory effect of TNFα and ceramides on erythropoiesis while inhibiting induction of myelopoiesis. In contrast, bafilomycin A1, but not siRNA against Atg5, induced myeloid differentiation, while both impaired erythropoiesis. We demonstrate here that the TNFα/neutral SMase/ceramide pathway inhibits erythropoiesis to induce myelopoiesis via modulation of a hematopoietic TF/miR network and inhibition of late steps of autophagy. Altogether, our results reveal an essential role of autophagy in erythroid vs. myeloid differentiation.

## Introduction

Inflammatory cytokines cause anemia of cancer and chronic inflammatory diseases with consequences for the quality of life and lifespan. Cytokines like tumor necrosis factor-α (TNFα) impair erythroid differentiation and perturbate hematopoietic homeostasis [1–5]. A network of receptors, cell signaling cascades, transcription factors (TFs), and microRNAs (miRs) time-dependently regulate hematopoiesis [6, 7]. TFs and miRs modulate genes involved in myeloid commitment. Major TF GATA-1 regulates erythropoiesis by modulating the negative regulator GATA-2 [8] and the erythroid miR-144/451 gene cluster [9]. Conversely, myeloid regulator PU.1 prevents erythropoiesis by binding to GATA-1. The GATA-1/PU.1 antagonism determines erythroid vs. myeloid cell fate [10]. Furthermore, PU.1 regulates miR-146a, miR-155, and miR-223 genes associated with inhibition of erythropoiesis and granulomonocytic development [11–13].

Erythrocytic but not granulomonocytic differentiation requires autophagic clearance of cytoplasmic organelles and expulsion of the nucleus [14–18]. Autophagosome formation requires the ULK1-Atg13-FIP200 complex [19]. This complex is inhibited by activation of the PI3K/AKT/mTOR pathway, which prevents Atg13^S355^ activation through ULK1^S758^ phosphorylation [20]. Vesicle nucleation involves Beclin-1 interacting with Rubicon (RUN domain protein as Beclin-1-interacting and cysteine-rich containing), a suppressor of autophagy [21]. Autophagosome formation requires Atg12/Atg5/Atg16 complex formation and lipidation of LC3 by Atg3/Atg7 [22]. Autophagosomes then fuse with endosomes/lysosomes triggering organelle and protein degradation including p62/SQSTM1 cargo [23].

TNFα-stimulated nuclear factor-κB and p38MAPK pathways regulate the inhibition of erythroid gene expression and differentiation [4, 24]. TNFα also activates neutral sphingomyelinase (nSMase) through a nSMase activation domain within the cytoplasmic region of the 55 kDa TNF receptor. TNF receptor internalization also leads to acid (a)SMase activation [25]. SMases subsequently hydrolyze sphingomyelin to ceramides [26–28], which regulate inflammation, differentiation, proliferation, apoptosis, senescence, and autophagy [29, 30]. Ceramides are metabolized to sphingosine and sphingosine-1-phosphate (S1P) by ceramidase and sphingosine kinase-1 (SphK1). Ceramide and S1P play antagonistic roles in various cellular processes forming the “sphingolipid rheostat” [31].

As the regulatory role of ceramides remains unknown in TNFα-mediated impairment of erythropoiesis, we used here an erythropoiesis model based on CD34^+^/hematopoietic stem progenitor cells (CD34/HSPCs) from umbilical cord blood (UCB) of healthy human donors. Altogether, we describe a novel TNFα/nSMase/ceramide pathway that inhibits erythropoiesis and promotes myeloid differentiation by modulating a TF/miR hematopoietic micro-network and by inhibiting autophagy.

## Results

### The TNFα/nSMase/ceramide pathway downregulates erythroid differentiation

**Fig. 1:**
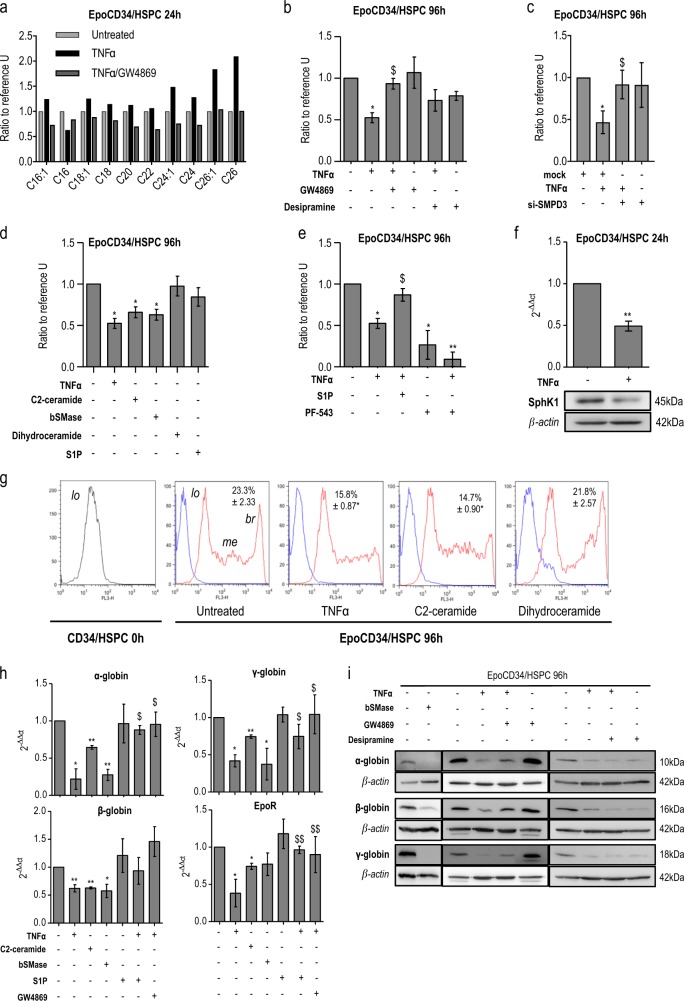
**a** Mass spectrometry analysis of C16-, C16:1-, C18-, C18:1-, C20-, C22-, C24-, C24:1-, C26-, and C26:1-ceramide in EpoCD34/HSPCs treated with TNFα and GW4869 for 24 h. Ceramide species were quantified by ESI-MS/MS and results were reported to the number of cells. EpoCD34/HSPCs (Epo: 2 U/mL) were untreated (U) or treated as indicated with TNFα, GW4869, desipramine, C2 ceramide, bSMase, dihydroceramide, sphingosine-1-phosphate (S1P), or PF-543 or transfected with si-SMPD3. **b** Benzidine staining assays with sphingomyelinase inhibitors at 96 h. **c** Benzidine staining assays with si-SMPD3 at 96 h. **d** Comparison of TNFα effect on benzidine staining assays at 96 h with ceramides and its derivatives. **e** Benzidine staining assays with S1P and PF-543 at 96 h. **f** Real-Time PCR and Western blot analysis of SphK1 expression at 24 h. **g** Flow cytometry analysis of GPA expression. GPA-expressing populations were identified as low (lo), medium (me), and bright (br). Mean of percentage ± SD of GPA^bright^ cells is indicated in each quadrant. **h** Real-time PCR analysis of globins and EpoR expression at 96 h. **i** Western blot analysis of globin's expression at 96 h. Bars represent the mean ± SD of four independent experiments except for **f** with three independent experiments. One-way ANOVA with repeated measures followed by Dunnett’s post hoc test show the statistical significance *^,$^*P* < 0.05, **^,$$^*P* < 0.01 (*: treatment vs. untreated; $: treatment vs. TNFα). A Student’s *t* test was performed for **f**. β-Actin expression was used in Western blot analyses as an internal control and was revealed each time on the same membrane than one or several proteins of interest

We initially assessed the ability of TNFα and GW4869 to modulate ceramide synthesis in erythropoietin (Epo)-treated CD34^+^/HSPCs (EpoCD34/HSPCs) by electrospray ionization-tandem mass spectrometry (ESI-MS/MS). Results showed that TNFα increased ceramide formation and particularly long-chain ceramide C24 and C24:1 after 24 h of treatment, while GW4869 prevented the effect of TNFα (Fig. 1a and Supplementary table S1). MS analysis and fluorescent immunostaining assays confirmed the rapid activation of ceramide production as early as 2 min (Supplementary Figure S1).

**Fig. 2:**
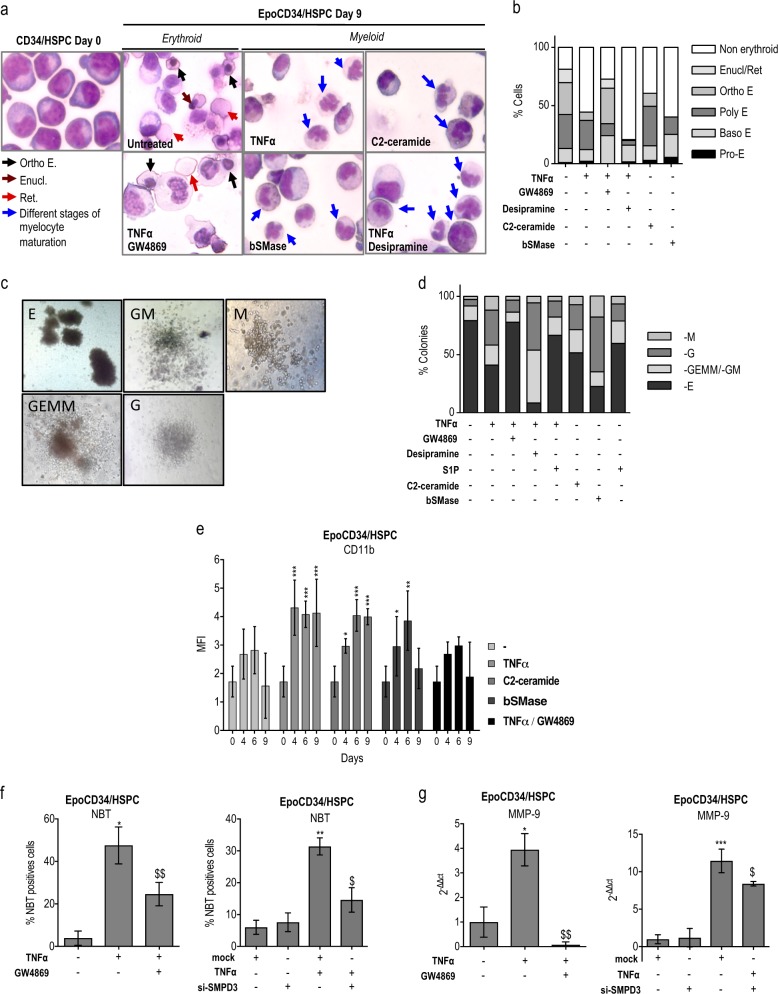

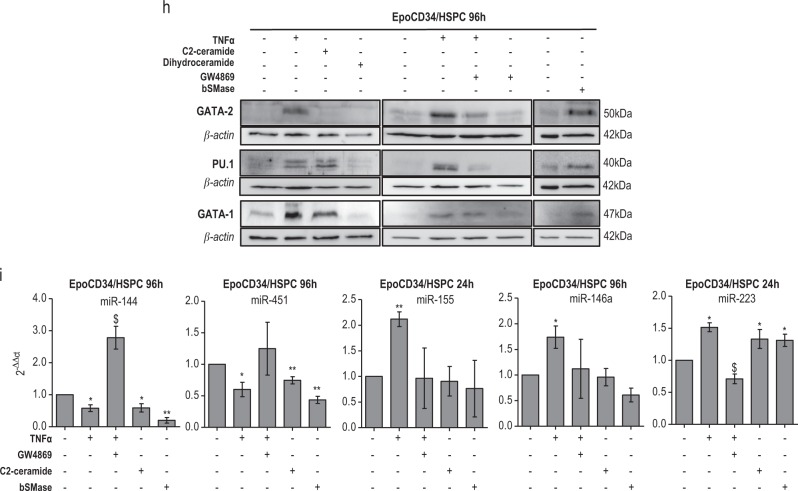
Impact of TNFα/SMase/ceramide pathway on EpoCD34/HSPCs differentiation. **a** May–Grünwald–Giemsa staining of CD34/HSPCs at day 0 and EpoCD34/HSPCs at day 9 of treatment observed with a ×40 objective. Each picture is representative of three independent experiments. Enucl/Ret: enucleation/reticulocyte; E: erythroblast; Ortho: orthochromatophilic. **b** Representation of the different cell types distribution on MGG-stained slides. **c** Microscope observation (×20) of distinctive morphological aspects of hematopoietic colonies obtained after EpoCD34/HSPCs treatments at day 14. E: erythroid; GM: granulocyte–monocyte; GEMM: granulocyte–erythrocyte–monocyte–megakaryocyte; G: granulocyte; M: monocyte/macrophage. **d** Representation of the different colonies distribution in colony formation assays of EpoCD34/HSPCs treatments at day 14. **e** Flow cytometry analysis of CD11b expression in EpoCD34/HSPCs from day 0 to 9 of culture. **f** NBT staining with GW4869 and si-SMPD3 at 96 h. **g** Real-time PCR analysis of *MMP-9* expression with GW4869 and si-SMPD3 at 96 h. **h** Western blot analysis of GATA-1, GATA-2, and PU.1 in EpoCD34/HSPCs after 96 h of treatment. β-Actin expression was used in Western blot analyses as an internal control and was revealed each time on the same membrane than one or several proteins of interest. **i** Real-time PCR analysis of miR-144, -451, -155, -146a, and -223 expression levels in EpoCD34/HSPCs. Bars represent the mean ± SD of five independent experiments. One-way ANOVA with repeated measures followed by Dunnett’s post hoc test or two-way ANOVA followed by Holm–Sidak’s post hoc test show the statistical significance *^,$^*P* < 0.05, **P < 0.01, ***P < 0.001 (*: treatment vs. untreated, $: treatment vs. TNFα)

To study the implication of the sphingolipid metabolism in TNFα-mediated inhibition of erythropoiesis, cell hemoglobinization was assessed by benzidine staining in EpoCD34/HSPCs. Results showed that TNFα inhibited hemoglobinization by 50% and that GW4869 treatment prevented this inhibition, whereas desipramine had no significant effect (Fig. 1b). Knock down of nSMase in EpoCD34/HSPCs by *SMPD3*-specific small interfering RNA (siRNA) (si-SMPD3) did not affect cell viability and allowed inhibition of nSMase messenger RNA (mRNA) and protein expression (Supplementary Figure S2). Results showed that the knock down of nSMase expression was able to restore the amount of hemoglobin-producing cells (Fig. 1c). The effect of ceramide production was then mimicked by treating EpoCD34/HSPCs with synthetic C2- and C6-ceramide, bacterial SMase (bSMase), and the bioactive sphingolipids derived from ceramide C2-dihydroceramide (dihydroceramide) and S1P. Like TNFα, C2- and C6-ceramide as well as bSMase prevented hemoglobinization of EpoCD34/HSPCs by 40% at 96 h, while dihydroceramide and S1P remained without effect (Fig. 1d and Supplementary Figure S3a). Nevertheless, S1P prevented TNFα-mediated inhibition of hemoglobinization, which was increased by 35%. Moreover, the inhibition of S1P synthesis by SphK1 inhibitor PF-543 reduced hemoglobinization by 75% and enhanced the effect of TNFα (Fig. 1e). Accordingly, TNFα inhibited SphK1 mRNA and protein expression, suggesting that inhibition of S1P production by TNFα contributed to impairment of erythropoiesis (Fig. 1f). Results showed that Epo induced three cell populations differentially expressing GPA: GPA^low^, GPA^medium^, and GPA^bright^ corresponding to increasing erythroid differentiation. GPA^low^ population was only present at 0 h. Treatment of EpoCD34/HSPCs with TNFα or C2-ceramide for 96 h reduced the formation of the GPA^bright^ population from 23 to 15%, while dihydroceramide had no effect (Fig. 1g). Furthermore, TNFα, C2-ceramide, and bSMase strongly reduced both mRNA and protein expression of erythroid markers (globins and EpoR) (Fig. 1h, j). Similarly, C6-ceramide decreased globin expression (Supplementary Figure S3b). In contrast, S1P and GW4869 prevented this effect while desipramine did not. To further investigate the impact of the TNFα/ceramide pathway on erythropoiesis, May–Grünwald–Giemsa (MGG) staining assays were performed after 9 days. Results showed abundant orthochromatophilic erythroblasts, enucleation pictures, and reticulocytes in EpoCD34/HSPCs at day 9 compared to day 0 (Fig. 2a). TNFα, C2-ceramide, and bSMase decreased the total number of erythroid cells by 55, 40, and 60%, respectively. Orthochromatophilic erythroblasts represented only 7 and 11% after TNFα and C2-ceramide treatment, respectively. bSMase completely abolished their production. Moreover, no reticulocyte or enucleation was identified under those conditions. GW4869 prevented the effect of TNFα with the restoration of 75% of erythroid cells at all maturation stages. Conversely, desipramine reinforced the effect of TNFα (Fig. 2b).

Our results support a fundamental role for the nSMase/ceramide pathway in TNFα-mediated impairment of erythropoiesis. Furthermore, TNFα perturbates the sphingolipid rheostat by inhibiting SphK1 expression leading to ceramide accumulation, exacerbating erythropoiesis inhibition.

### The TNFα/nSMase/ceramide pathway converts erythropoiesis to myelopoiesis

Considering that TNFα and ceramides reduced erythroid differentiation in EpoCD34/HSPCs without inducing cell death (Supplementary Figure S3c and S4), we assessed the fate of the remaining non-erythroid cell population. Following TNFα, C2-ceramide, and bSMase treatments, MGG staining revealed different stages of myeloid maturation including polymorphonuclear neutrophils (Fig. 2a, b). Colony formation assays with EpoCD34/HSPCs confirmed the distribution of cellular differentiation. Assays displayed 79% of burst-forming unit-erythroid/colony-forming unit-erythroid (BFU-E/CFU-E) colonies, while the remaining colonies were represented by CFU–granulocyte–erythrocyte–monocyte–megakaryocyte (GEMM), CFU-GM, CFU-G, and CFU-M after 14 days (Fig. 2c, d). Following TNFα, C2-ceramide, and bSMase treatments, myeloid colonies CFU-G/CFU-M increased by 5-, 3.5- and 8-fold, respectively, concomitant with a decrease of BFU-E/CFU-E colonies reaching 41, 51 and 22.5%, respectively (Fig. 2d). Moreover, S1P treatment prevented the decrease of BFU-E formation. Importantly, GW4869 efficiently prevented the effect of TNFα by restoring the score of BFU-E/CFU-E to 78%, whereas desipramine reduced BFU-E/CFU-E formation concurrently to an enhancement of myeloid subpopulations (Fig. 2d and Supplementary Table S2). TNFα and ceramides significantly increased CD11b expression from 96 h and GW4869 prevented the induction (Fig. 2e and Supplementary Figure S3d). Furthermore, nSMase inhibition by GW4869 and knock down by si-SMPD3 also prevented TNFα-induced neutrophil differentiation as shown by nitro blue tetrazolium (NBT) staining and the downregulation of matrix metalloproteinase (*MMP*)*-9* gene (Fig. 2f, g and Supplementary Figure S5). Our results show that the TNFα/nSMase/ceramide pathway inhibits erythropoiesis concomitantly with granulomonocytic differentiation.

### Myeloid differentiation and inhibition of erythropoiesis correlate with the modulation of related TFs and miRs

We then considered the conversion of erythroid to myeloid differentiation in EpoCD34/HSPCs treated by TNFα and ceramides. The effect of the TNFα/ceramide pathway was analyzed via the expression of key regulators of myelopoiesis and erythropoiesis, including TFs PU.1, GATA-1, and GATA-2, as well as related miRs at different time points (Fig. 2h, i and Supplementary Figure S6). TF expression was not affected after 24 h by TNFα (Supplementary Figure S6). At 96 h, expression of the erythropoiesis repressors GATA-2 and PU.1 were induced in TNFα-treated cells in a nSMase-dependent manner as shown in GW4869-co-treated cells. PU.1 was also up-regulated by C2-ceramide and bSMase, in correlation with both the inhibitory effect on erythropoiesis and the induction of myelopoiesis. GATA-2 expression was induced by bSMase treatment, while C2-ceramide had no effect. Even though cells were committed towards the myeloid lineage and erythroid-specific genes were downregulated, GATA-1 remained expressed after 96 h of TNFα treatment, in agreement with previous data [2]. Similarly, C2-ceramide and bSMase sustained GATA-1 expression, whereas dihydroceramide had no effect (Fig. 2h).

In agreement with the impairment of erythropoiesis and the myeloid commitment, hematopoiesis-related miRs were also modulated. TNFα, C2-ceramide, and bSMase inhibited the expression of the positive regulators of erythropoiesis miR-144 and miR-451, which are regulated by GATA-1. Accordingly, GW4869 prevented TNFα-mediated inhibition of these miRs. TNFα induced the expression of the myeloid-specific miR-146a, miR-155, and miR-223, controlled by PU.1. Likewise, GW4869 prevented the upregulation of these miRs. However, C2-ceramide and bSMase did not affect miR-146a and miR-155, but induced the granulocytic miR-223 (Fig. 2i). Results suggested that the TNFα/nSMase/ceramide pathway modulates key regulators of both myeloid differentiation and erythropoiesis.

### The TNFα/nSMase/ceramide pathway impairs autophagy

Autophagy is critical for erythropoiesis and was reported as inversely correlated to neutrophil differentiation [17]. Thus, we assessed the capacity of the TNFα/nSMase/ceramide pathway to impair autophagy.

**Fig. 3:**
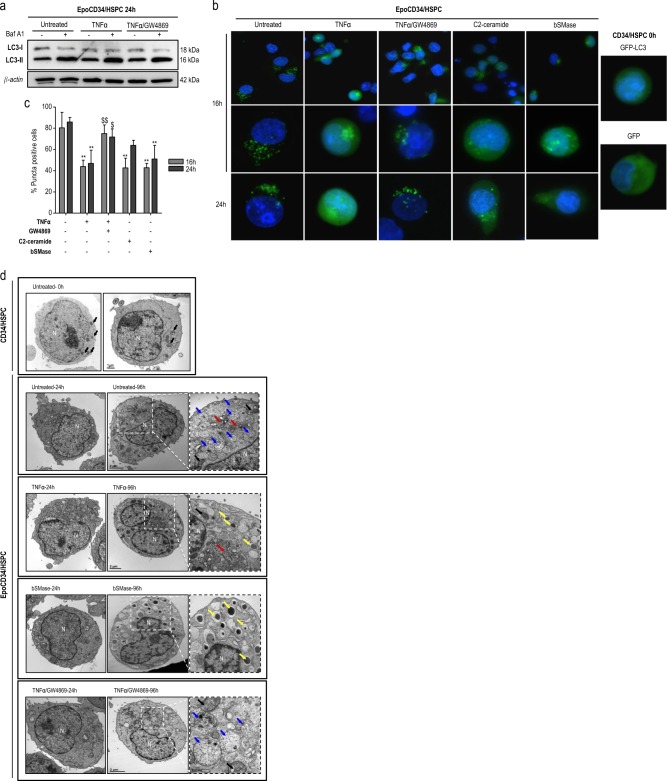
Impact of TNFα/SMase/ceramide pathway on EpoCD34/HSPCs autophagy. **a** Western blot analysis of LC3 conversion in EpoCD34/HSPCs treated as indicated for 24 h. BafA1: bafilomycin A1. β-Actin expression was used in Western blot analyses as an internal control. **b** Fluorescence microscopy observations of Epo-free or EpoCD34/HSPCs after nucleofection with pSelect-GFP-LC3 plasmid or with GFP control plasmid and treatments as indicated for 0, 16, and 24 h. Nuclei were stained with 4′,6-diamidino-2-phenylindole (DAPI). Two lower rows show magnified cells representative of the transfected population (×40); in row 1, cells are shown in a larger field at 24 h (×20). **c** Quantification of transfected viable cells that display *punctae* pattern without diffuse signal. Bars represent the mean ± SD of three independent experiments. Two-way ANOVA followed by Holm–Sidak’s post hoc test show the statistical significance *^,$^*P* < 0.05, **^,$$^*P* < 0.01 (*: vs. untreated, $: vs. TNFα). **d** TEM pictures of untreated CD34/HSPCs at 0 h and of untreated or treated as indicated EpoCD34/HSPCs at 24 h and 96 h. N: nucleus; blue arrows: autophagosome/autolysosome, black arrows: mitochondria, red arrows: Golgi, yellow arrows: dense-core early azurophilic granules and secretion vesicles

The ability of Epo to induce an autophagic flux was first evaluated by analyzing LC3 conversion at 0, 24, 72, 96, and 120 h in the presence or absence of bafilomycin A1 (BafA1) by Western blot (Supplementary Figure S7) where the accumulation of LC3-II increased with time. To assess the effect of TNFα and GW4869 on Epo-mediated autophagy in CD34/HSPCs, we performed Western blot analysis of LC3 conversion. Results showed that in the presence of BafA1, LC3-II accumulated in all conditions including in untreated and TNFα-treated cells at 24 h (Fig. 3a). Therefore, we assessed the effect of TNFα, C2-ceramide, bSMase, and GW4869 on autophagy by transfecting CD34/HSPCs with a GFP-LC3 expression construct. Fluorescence microscopy allowed to quantify cells with GFP-LC3 *punctae* at 16 and 24 h visible in 80% of transfected EpoCD34/HSPCs vs. 3% in CD34/HSPCs (Fig. 3b, c). TNFα, C2-ceramide, and bSMase significantly reduced the percentage of cells displaying GFP-LC3 *punctae* by 50%. The inhibition of nSMase by GW4869 prevented the effect of TNFα as shown by the restoration of *punctae* formation. These results suggested that TNFα and ceramides inhibit autophagy in EpoCD34/HSPCs. To further confirm these results, we performed observations by transmission electron microscopy (TEM) (Fig. 3d). The typical phenotype of immature HSPCs could be observed at 0 h with a large nucleus and early structures of autophagy (phagophores), which remained visible at 24 h in EpoCD34/HSPCs whatever the treatment used. At 96 h, we observed abundant autophagosome/autolysosome accumulation in EpoCD34/HSPCs confirming activation of autophagy. Following TNFα and bSMase treatments, the absence of autophagosome/autolysosome accumulation suggested an impairment of autophagy. Moreover, dense-core early azurophilic granules in the Golgi region as well as the nuclear lobulation suggested a promyelocytic differentiation. Interestingly, GW4869 prevented the inhibition of autophagosome formation. We document here that the TNFα/ceramide pathway inhibits autophagy in EpoCD34/HSPCs concomitantly with inhibition of erythropoiesis.

### The TNFα/nSMase/ceramide pathway impairs autophagy via mTOR activation

**Fig. 4:**
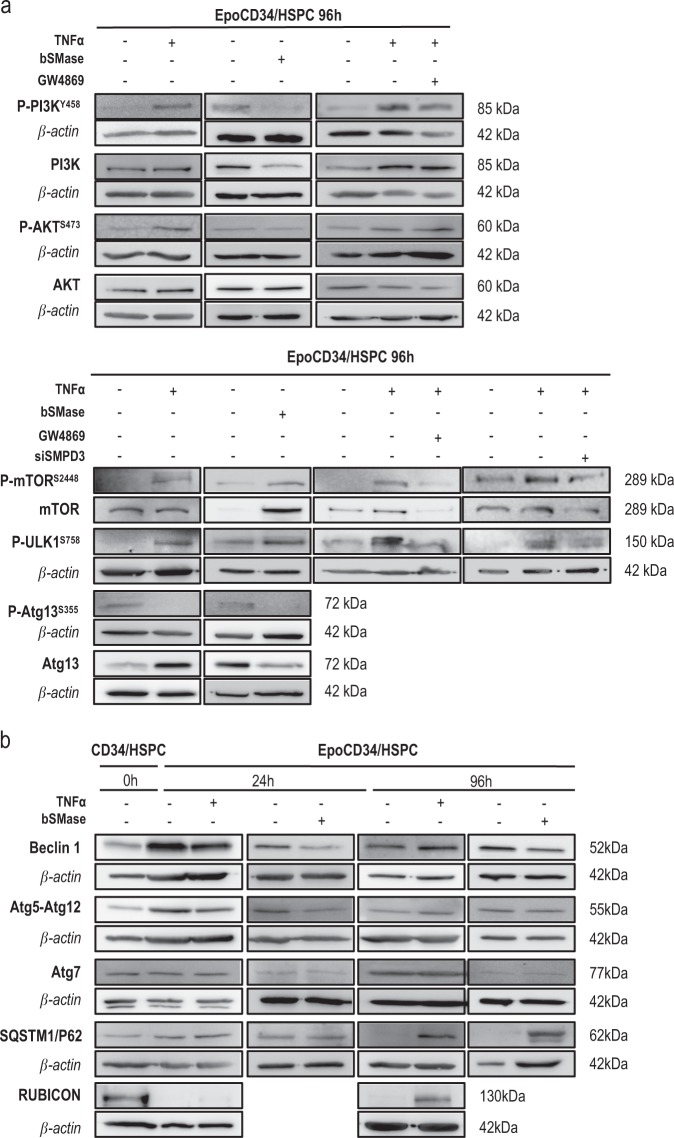
Western blot analysis of autophagy-related proteins expression in EpoCD34/HSPCs. **a** PI3K/AKT/mTOR/ULK1/Atg13 pathway of untreated or treated cells as indicated or transfected with si-SMPD3 at 96 h. **b** Beclin-1, Atg5-12, Atg7, p62/SQSTM1, and Rubicon in untreated CD34/HSPCs at 0 h and in EpoCD34/HSPCs untreated or treated as indicated at 24 and 96 h. β-Actin expression was used in Western blot analysis as an internal control and was revealed each time on the same membrane than one or several proteins of interest

We then investigated the mechanistic aspects of the inhibition of autophagy by the TNFα/nSMase/ceramide pathway. At 24 h, we observed that TNFα increased phosphorylation of PI3K and mTOR. The PI3K/AKT/mTOR/ULK1^S758^ pathway was fully activated after 96 h, leading to inhibition of Atg13^S355^ phosphorylation, thus affecting autophagy (Fig. 4a, Supplementary Figure S8). Treatments with bSMase also induced phosphorylation of mTOR^S2448^ and ULK1^S758^ and subsequent inhibition of Atg13^S355^ phosphorylation, but this effect was independent of PI3K/AKT pathway activation. Inhibition of nSMase in TNFα-treated cells through pharmacological or genetic inhibition with GW4869 or si-SMPD3 prevented the effect of TNFα on mTOR^S2448^/ ULK1^S758^ phosphorylation, supporting the implication of the nSMase pathway in autophagy impairment (Fig. 4a).

Analysis of autophagy-related proteins showed that Beclin-1 expression and Atg5-Atg12 complex formation was induced in Epo-treated CD34/HSPCs at 24 h compared to 0 h, while Rubicon expression was abolished. At 24 h, TNFα and bSMase induced an inhibition of Beclin-1 and Atg5-Atg12, which did not persist at 96 h. Atg7 was not affected. Interestingly, p62/SQSTM1 protein remained undetectable in untreated EpoCD34/HSPCs at 96 h, while it accumulated in TNFα- and bSMase-treated cells witnessing an inhibition of autophagy. Similarly, Rubicon expression was strongly induced at 96 h by TNFα treatment compared to untreated cells in agreement with autophagy inhibition (Fig. 4b). Activation of the mTOR pathway transduced the repressive potential of TNFα and ceramides on Epo-mediated physiological autophagy.

### Autophagy is required for TNFα/ceramide-mediated modulation of differentiation

**Fig. 5:**
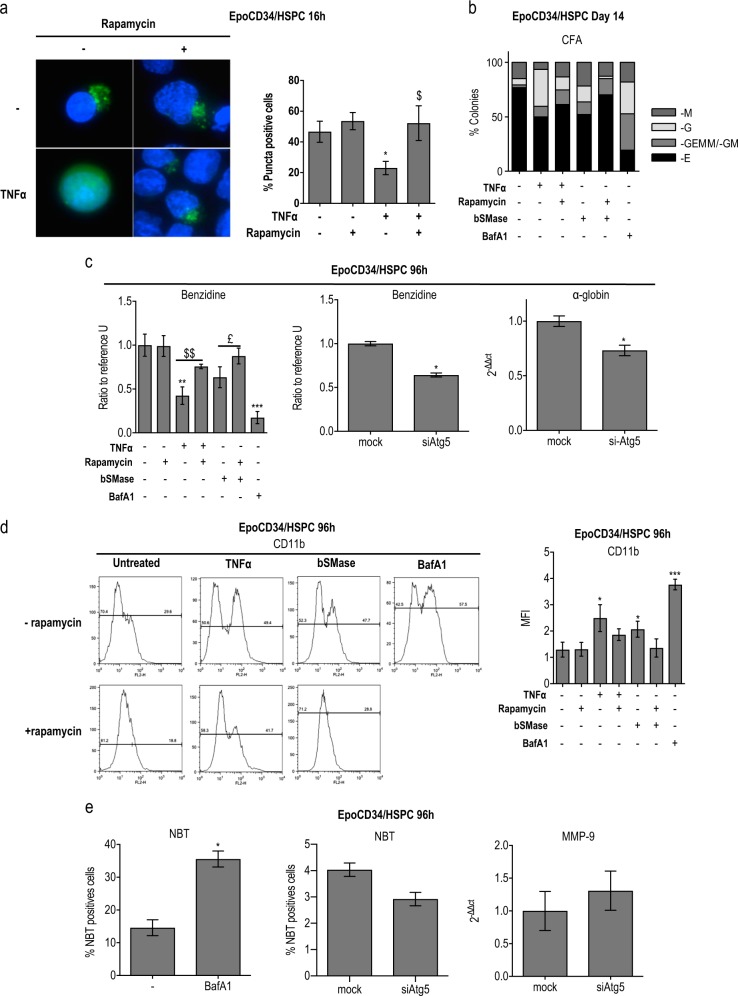
Impact of autophagy on erythroid vs. myeloid commitment. EpoCD34/HSPCs were treated with bafilomycin A1 (BafA1) and with rapamycin in the presence or absence of TNFα or bSMase during 96 h. **a** Fluorescence microscopy observation of EpoCD34/HSPCs after nucleofection with pSelect-GFP-LC3 plasmid and treatments with TNFα and/or rapamycin for 16 h. Nuclei were stained with 4′,6-diamidino-2-phenylindole (DAPI). Cells were observed with ×40 objective. Transfected viable cells that display *punctae* pattern without diffuse signal were quantified. Bars represent the mean ± SD of three independent experiments. Two-way ANOVA followed by Holm–Sidak’s post hoc test show the statistical significance *^,$^*P* < 0.05 (*: vs. untreated, $: vs. TNFα). **b** Colony formation assays at day 14. **c** Benzidine staining and real-time PCR analysis of α-globin mRNA expression in the presence of autophagy modulators at 96 h. **d** Flow cytometry analysis of CD11b expression in EpoCD34/HSPCs at 96 h. **e** NBT staining and real-time PCR analysis of MMP-9 mRNA expression with bafilomycin A1 (BafA1) and si-Atg5 at 96 h. Bars represent the mean ± SD of three or five independent experiments. One-way ANOVA with repeated measures followed by Dunnett’s post hoc test showed the statistical significance *^,£^*P* < 0.05, **^,$$^*P* < 0.01, ***P < 0.001 (*: vs. untreated, $: vs. TNFα, £: vs. bSMase)

We then assessed the requirement of autophagy in the effect of the TNFα/nSMase/ceramide pathway on CD34/HSPC differentiation. The efficiency of rapamycin as an mTOR inhibitor and autophagy inducer was first verified in our model under different conditions by GFP-LC3 transfection and fluorescent microscopy (Fig. 5a). We assessed the ability of the mTOR inhibitor rapamycin to restore autophagy in TNFα-treated cells by performing GFP-LC3 transfections in EpoCD34/HSPCs. Numeration of cells displaying *punctae* at 16 h by fluorescence microscopy showed that rapamycin restored *punctae* formation in TNFα-treated cells, verifying simultaneously the ability of rapamycin to induce autophagy in our model. Rapamycin had no significant effect in Epo-treated cells that already presented autophagic features.

We then analyzed the ability of rapamycin-induced autophagy to prevent inhibition of erythropoiesis in EpoCD34/HSPCs treated by TNFα or bSMase for 96 h. Rapamycin was able to restore erythroid differentiation in TNFα- and bSMase-treated cells as shown by CFU-E/BFU-E formation and the partial restoration of hemoglobin-producing cells (Fig. 5b, c). Results showed that rapamycin-induced autophagy in TNFα-treated cells correlated with induced erythroid differentiation. Concomitantly, the myeloid colonies and CD11b expression were reduced (Fig. 5b, d). Conversely, inhibition of the late stage of autophagy by BafA1 strongly decreased the amount of Epo-induced CFU-E/BFU-E formation as well as the percentage of hemoglobin-producing cells while favoring myeloid colonies (Fig. 5b–d). On the other hand, we set up CD34/HSPC transfection with siRNA against Atg5 (si-Atg5) and verified its efficiency on Atg5 expression (mRNA and protein) as well as its ability to inhibit autophagy by LC3 conversion analysis (Supplementary Figure S2). Interestingly, knock down of the early-step autophagy *Atg5* gene with si-Atg5 affected erythroid differentiation as shown by reduced benzidine staining and decreased α-globin mRNA expression (Fig. 5c). EpoCD34/HSPCs treated with BafA1 displayed an increase in granulocytic intracytoplasmic deposits of reduced formazan (NBT staining), whereas this marker and MMP-9 mRNA expression were not significantly affected in si-Atg5-transfected cells (Fig. 5e). Co-treatment of EpoCD34/HSPCs with TNFα or bSMase and rapamycin had no effect on cell viability and BafA1 reduced cell viability by 15% at 96 h (Supplementary Figure S4).

These results underlined that inhibition of erythropoiesis resulted from TNFα/ceramide-induced autophagy impairment. Besides, our data suggest that granulocyte differentiation depends on late autophagy inhibition since inhibition of the early-step regulator Atg5 had no effect on granulocyte differentiation.

## Discussion

**Fig. 6:**
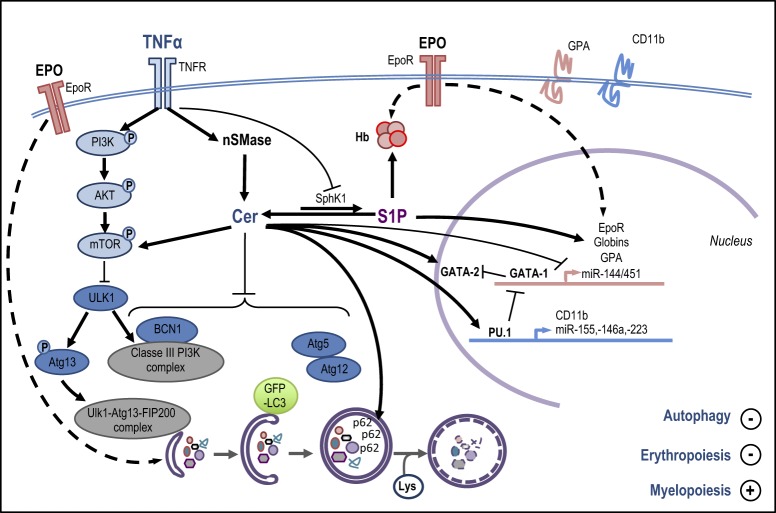
Erythropoietin (Epo) binds to the Epo receptor (EpoR) to activate erythroid-specific gene transcription (*EpoR*, *globins*, *glycophorin A* (GPA), and *miR-144/451*) by GATA-1, resulting in hemoglobin (Hb) production. Epo-induced erythropoiesis is concomitant with autophagy flux induction. TNFα binds its receptor TNFR to activate neutral sphingomyelinase (nSMase) catalyzing ceramide (Cer) production. Ceramides inhibit erythroid gene expression and induce GATA-2, PU.1, CD11b, and miR-155, -146a, and -223 expression. TNFα inhibits SphK1 leading to a ceramide/S1P unbalanced reaction. S1P has a positive effect on Hb production and erythroid marker expression. TNFα and ceramides inhibit autophagy. TNFα phosphorylates mTOR (P-mTOR) via the PI3K/AKT pathway, while ceramide activates mTOR through a different mechanism. P-mTOR leads to ULK1 inhibition and the subsequent Atg13 dephosphorylation. TNFα and ceramides inhibit Beclin-1 (BCN1) expression, the formation of the Atg5-Atg12 complex, GFP-LC3 *punctae*, and autophagosomes, and induce p62/SQSTM1 accumulation. Together, the effects triggered by the TNFα/neutral SMase/ceramide pathway at the molecular level lead to autophagy and erythropoiesis inhibition and myelopoiesis induction

The molecular mechanisms describing how ceramides regulate TNFα-mediated conversion of erythropoiesis to myelopoiesis in EpoCD34/HSPCs through inhibition of autophagy and modulation of specific TFs/miRs are summarized Fig. 6.

We show that exogenous C2- and C6-ceramide and bSMase-induced endogenous ceramides prevented the expression of erythroid markers, erythropoiesis features, and miR-144/451 expression in EpoCD34/HSPCs similarly to TNFα. Moreover, nSMase inhibitor GW4869 and siRNA targeting nSMase showed that the TNFα/nSMase pathway regulates the inhibition of erythropoiesis. Ceramides were previously described to inhibit erythropoiesis, induce apoptosis, and reduce CFU-E formation from peripheral blood mononuclear cells [32].

Importantly, whatever the treatment used here, cell viability remained unaffected, excluding involvement of apoptosis in erythropoiesis inhibition.

Besides ceramides, other derivatives such as S1P are involved in cell differentiation and autophagy [30]. Blockade of S1P formation by inhibiting SphK1 prevented erythroid differentiation and enhanced the effect of TNFα, supporting a positive role for S1P in erythropoiesis. Interestingly, TNFα also inhibited the expression of SphK1, which is regulated by GATA-1 [33]. Similarly, treatments with S1P prevented the inhibition of erythropoiesis by TNFα in agreement with its antagonistic effect on ceramides [31]. On the other hand, ceramidases are unable to generate sphingosine upon hydrolysis of C2-ceramide, so that this intermediate metabolite is likely not involved in erythropoiesis inhibition [34]. Altogether, these results hint at ceramides as the main bioactive sphingolipids involved in TNFα-mediated inhibition of erythropoiesis.

We show that the TNFα/nSMase/ceramide pathway leads both to the inhibition of Epo-activated erythropoiesis and the activation of myeloid differentiation. In agreement with our results, TNFα and Vitamin D3, an inducer of granulocyte differentiation, were reported to activate ceramide metabolism in HL60 cells [35, 36]. Remarkably, granulopoiesis was enhanced by the PI3K/AKT/mTOR cascade and by overexpression of AKT in murine CD34/HSPCs, whereas an inhibition of AKT led to the absence of differentiation [37–39]. Here, TNFα-mediated activation of PI3K/AKT/mTOR and p62/SQSTM1 accumulation correlated with granulocytic differentiation [40].

Results show that TNFα and ceramides induce granulopoiesis despite the presence of Epo, suggesting a role for the TNFα/nSMase/ceramide pathway in cell fate determination. According to their interaction and cooperation modalities, TFs GATA-1, GATA-2, and PU.1 are essential parameters for hematopoietic cell commitment [41–43]. Modulation of these TFs is correlated with TNFα/ceramide-mediated impairment of erythropoiesis and activation of myelopoiesis. Despite the active role of GATA-1 in erythroid gene transcription, our results also showed increased GATA-1 expression after TNFα, C2-ceramide, and bSMase treatments in agreement with reports describing that GATA-1 overexpression inhibited erythropoiesis in mouse erythroblasts culminating in lethal anemia [44]. Besides, our results correlate with an inhibition of GATA-1 activity. Indeed, GATA-1 target genes, including erythroid-specific genes, *miR-144/451* and *SphK1*, are downregulated. Furthermore, impairment of GATA-1 activity can be explained by both GATA-2 and PU.1 overexpression. Indeed, active GATA-1 inhibits GATA-2 transcription, while overexpression of GATA-2 inhibits erythropoiesis [8, 45]. Moreover, GATA-2 activates GATA-1 transcription. As a result, GATA-2 overexpression in TNFα- and ceramide-treated cells leads to GATA-1 overexpression in agreement with both GATA-1 inactivation and inhibition of erythropoiesis. Besides, loss of GATA-1 activity correlates with induction of the myeloid TF PU.1, which inhibits GATA-1 through physical interaction [10, 46]. We previously reported that TNFα promoted the GATA-1/PU.1 inhibitory complex in EpoCD34/HSPCs [2].

Obviously, increased PU.1 levels not only coincided with erythropoiesis inhibition but also triggered myelopoiesis with CD11b, miR-223, miR-146a, and miR-155 upregulation [47]. TNFα and ceramides induced miR-223, while only TNFα induced miR-146a and miR-155. In agreement with our results, these miRs are also inhibitors of erythropoiesis [13, 48–50].

Autophagy is required for physiological erythropoiesis to eliminate organelles. Knock out of the autophagic regulators, ULK1, Atg4, Atg7, or FIP200, or mitophagy-specific receptor, NIX, inhibited erythropoiesis leading to anemia in mice while inducing myeloid expansion [16, 18, 51–53]. In contrast to erythropoiesis, autophagy is not essential for neutrophil differentiation [17]. At 24 h, Beclin-1 induction, Atg5/Atg12 complex formation, the presence of GFP-LC3 *punctae* but the absence of mature autophagosomes, and sustained p62/SQSTM1 protein expression suggested that autophagy was initiated but not completed in EpoCD34/HSPCs. Nevertheless, this early autophagic flux was inhibited by TNFα and bSMase. Western blot analysis showed that TNFα and GW4869 did not change endogenous LC3-II levels. Besides its presence in autophagic membranes, LC3-II can be generated in an autophagy-independent manner and a significant amount of LC3-II was detectable after autophagy inhibition [54]. At 96 h, EpoCD34/HSPCs presented autophagic features, while TNFα and bSMase inhibited autophagy. At the molecular level, the PI3K(p85^Y458^)/AKT^S473^/mTOR^S2448^/ULK1^S758^ pathway was activated by TNFα. Interestingly, bSMase activated the mTOR^S2448^/ULK1^S758^ pathway independently of the PI3K(p85^Y458^)/AKT^S473^ pathway. This was supported by the fact that GW4869 was unable to prevent TNFα-induced PI3K/AKT activation. In agreement with our results, autophagy was inhibited in TNFα-treated Caco-2 cells, and TNFα acted as an activator of mTOR via IκΒ-kinases involving or not AKT in different cancer cell models [55, 56]. Atg13^S355^ phosphorylation was inhibited by TNFα and bSMase, in agreement with ULK1 inactivation, supporting an inhibitory effect on autophagy induction. Our results also indicate that ceramides and TNFα may affect autophagy through distinct mechanisms in EpoCD34/HSPCs. Another evidence of autophagy inhibition was the expression of Rubicon after 96 h of TNFα treatment while it was absent in untreated EpoCD34/HSPCs. Rubicon suppresses autophagosome maturation through physical interaction between its RUN domain and the catalytic subunit of hVps34. Indeed, ectopically expressed Rubicon reduced autophagosome maturation in U2OS cells, while Rubicon depletion accelerated p62/SQSTM1 protein degradation [21]. Our results also showed that TNFα and bSMase induced an accumulation of p62/SQSTM1 in EpoCD34/HSPCs after 96 h in correlation with autophagy inhibition [57]. Our results obtained by TEM, GFP-LC3 transfection, investigation of signaling pathways, and autophagy protein expression levels demonstrated that TNFα and ceramides affect autophagy in EpoCD34/HSPCs. The inhibition of early and late steps of autophagy by si-Atg5 and BafA1 treatment, respectively, led to inhibition of erythropoiesis of EpoCD34/HSPCs comparable to a treatment by TNFα and bSMase. Accordingly, the mTOR inhibitor rapamycin was able to prevent the effect of TNFα and bSMase by inducing both autophagy and erythropoiesis.

Our results demonstrate that the impairment of erythropoiesis results from an inhibition of autophagy in TNFα- and ceramide-treated CD34/HSPCs. Granulocytic differentiation was prevented by rapamycin in TNFα-treated cells, while the late-step inhibitor of autophagy BafA1 induced differentiation underlining the role of autophagy inhibition in this differentiation pathway. Nevertheless, *Atg5* knockdown was not sufficient to induce granulocytic differentiation in the presence of Epo, suggesting that erythropoiesis impairment is not the direct cause of myeloid differentiation. Altogether, these results underline the key role of autophagy via mTOR signaling in the modulation of EpoCD34/HSPC differentiation by TNFα, nSMase, and ceramides.

Overall, our results demonstrate that the TNFα/nSMase/ceramide pathway interferes with hematopoietic homeostasis providing new insights into hematopoietic cell fate determination as well as in inflammation-related anemia. Inhibition of erythropoiesis and induction of myelopoiesis by the TNFα/nSMase/ceramide pathway is supported by the modulation of key regulators of hematopoiesis. Furthermore, autophagy inhibition is a key event in impairment of erythropoiesis and induction of granulocytic differentiation in EpoCD34/HSPCs.

## Materials and methods

### Antibodies and reagents

Desmethylimipramine (desipramine), *N*,*N*′-Bis[4-(4,5-dihydro-1*H*-imidazol-2-yl)phenyl]-3,3′-*p*-phenylene-bis-acrylamide dihydrochloride (GW4869), SMase from *Staphylococcus aureus*, C2 dihydroceramide, S1P, and BafA1 were purchased from Sigma-Aldrich (Bornem, Belgium), C2-ceramide from Santa Cruz Biotechnology (Boechout, Belgium) and C6-ceramide from Enzo Life Sciences (Antwerp, Belgium). Human recombinant TNFα was kindly provided by Pr. Athanase Visvikis (Université de Lorraine, Nancy, France). Stem cell factor (SCF) and interleukin (IL)-3 were purchased from ReliaTech (Wolfenbüttel, Germany). The erythroid differentiation is induced with human recombinant erythropoietin (Epoietin beta, Neorecormon Roche, Grenzach-Whylen, Germany). The primary antibodies used were directed against: GATA-1, GATA-2, PU.1, α-, β-, and γ-globin (Santa Cruz Biotechnology), SphK1, PI3K, P-PI3K^Y458^, AKT, P-AKT^S473^, P-ULK1^S758^, mTOR, P-mTOR^S2448^, Atg7, Atg5-Atg12, Atg13, P-Atg13^S355^, Beclin-1, p62/SQSTM1 (Cell Signaling, Leiden, The Netherlands), LC3 (Sigma), Rubicon (Abcam, Cambridge, UK), and ceramide *MID 15B4* (Enzo Life Sciences, Antwerpen, Belgium), and the antibodies used for flow cytometry (CD235a/GPA, CD11b) were purchased from BD BioSciences (Erembodegem, Belgium).

### CD34^+^/HSPC isolation, culture, and treatment

UCB is kindly provided by Clinique Bohler, Luxembourg and Unité de Thérapie Cellulaire et banque de Tissus (UTCT), Nancy, France. It is collected after normal deliveries with a donor’s written informed consent in agreement with the National Committee of Research Ethics in Luxembourg. CD34^+^/HSPCs were purified as previously described [2, 58]. Briefly, mononuclear cells were collected using Ficoll^®^ (GE Healthcare, Roosendaal, The Netherlands) density gradient medium. Then, CD34^+^ cells were selected by magnetic cell sorting following the manufacturer’s instructions (Miltenyi Biotec, Utrecht, The Netherlands). CD34^+^ cells obtained were then cultivated in serum-free condition in Stem Line Medium II (Sigma) supplemented with l-glutamine, penicillin/streptomycin/amphotericin B (Lonza, Verviers, Belgium), IL-3 (10 ng/mL), and SCF (50 ng/mL) for 3 days (Supplementary Figure S10). At days 0, 4, and 6, epoietin beta (Epo) (2 U/mL) was added 1 h after the additon of TNFα (20 ng/mL), C2-ceramide (30 µM), C2-dihydroceramide (30 µM), or the bSMase (0.5 U/mL). The Smase inhibitors GW4869 (20 µM) and desipramine (10 µM) were added 1 h before TNFα treatment. Cultures were maintained at 2 × 10^5^ cells/mL. IL-3 was removed from the medium on day 4 and SCF on day 6. Cell number and viability were assessed using Trypan blue dye exclusion assay, and all cells were cultured in an incubator at 37 °C, 95% humidity, and 5% CO_2_. Cell viability was confirmed by Annexin V/propidium iodide staining and fluorescence-activated cell sorter (FACS) analysis according to the manufacturer's protocol (BD Biosciences).

### Profiling of ceramides by ESI-MS/MS

Ceramides were extracted according to the method by Kyrklund [59] in the presence of deuterium-labeled standards (*N*-heptadecanoyl-d-erythro-sphingosine (C17:0-ceramide); *N*-palmitoyl(d31)-d-*erythro*-sphingosylphosphorylcholine (C16:0D31SM) from Avanti Polar Lipids, Alabama, USA). Briefly, total lipids were extracted in chloroform:methanol (1:2 (v/v)). Sphingolipids were isolated by saponification after the addition of internal standards, and fractionated and desalted using reverse-phase Bond Elut C18 columns. The dry extracts were kept at −20 °C until MS/MS analysis. Samples were homogenized in chloroform:methanol (1:2 (v/v)) and analyzed by direct flow injection on a triple-quadrupole mass spectrometer (API 4500 QTRAP MS/MS; Sciex Applied Biosystems, Toronto, ON, Canada) in the positive ionization mode using the multiple reaction monitoring (MRM) method. Ceramide species were measured with a flow rate of 200 μl/min (analysis time of 3 min). The quantity of each molecular species was calculated from the ratio of its signal to that of the corresponding internal standard and normalized to the amount of cells.

### Assessment of cell differentiation

Erythroid differentiation was assessed by evaluating the rate of hemoglobin-producing cells after benzidine staining as previously described [2]. Granulocytic differentiation was assessed by evaluating the rate of formazan deposit-producing cells upon activation with PMA (phorbol 12-myristate 13-acetate) by NBT assay. Differentiation status of CD34^+^ cells was determined by microscopy analysis of the cell morphology (Leica DM2000 microscope, Lecuit, Howald, Luxembourg) at day 9 after MGG staining (Merck, Leuven, Belgium) and by flow cytometry analysis (FACSCalibur, Beckton-Dickinson, San Jose, CA, USA) of CD235a/GPA erythroid markers and CD11b granulomonocytic markers at days 0, 4, 6, and 9. Results were analyzed using the FlowJo software (version 8.8.7, Tree Star, Ashland, OR, USA) and statistical analysis was based on 10,000 events per sample. Colony formation assay was performed by seeding CD34^+^ cells in Methocult H4230 (StemCell Technologies, Vancouver, BC, Canada) supplemented with l-glutamine, penicillin/streptomycin/amphotericin B (Lonza), IL-3 (10 ng/mL), and SCF (50 ng/mL) and treated in the same conditions than suspension cultures. Colonies were counted after 14 days according to featured differentiation types and images were collected with a Leica DMIRB microscope.

### CD34/HSPC transfection

CD34/HSPCs were transfected with nSMase 2 (*smpd3*) pre-designed siRNA (Thermofisher, Erembodegen, Belgium) and Atg5 siRNA (Qiagen, Venlo, The Netherlands). Cells were transfected using the Human CD34 cell Nucleofector^®^ Kit (Lonza, Westburg, Leusden, The Netherlands). Transfection efficiency was assessed by Western blot and real-time PCR analysis of nSMase 2 and Atg5 expression at 24 h.

CD34/HSPCs were transfected at 0 h with pSELECT-GFP-hLC3 plasmid (Invivogen, Toulouse, France) using the Human CD34 cell Nucleofector^®^ Kit according to the manufacturer’s instructions. After 4 h, cells were treated, and the transfection efficiency of GFP-LC3, as well as the effect of the different treatments on *punctae* formation, were assessed by fluorescence microscopy after 16 and 24 h (Olympus, Aartselaar, Belgium). Nuclei were stained with 4′,6-diamidino-2-phenylindole (DAPI).

### Transmission electron microscopy

Cell samples were fixed for 1 h in 2.5% glutaraldehyde (Euromedex, Mundolsheim, France), diluted in 0.1 M cacodylate buffer (Euromedex), pH 7.2, and washed twice in cacodylate buffer. Cells were then postfixed 2 h in 2% osmium tetraoxide, washed with distilled water, and stained with 0.5% uranyl acetate overnight. Samples were then dehydrated in successive ethanol washes, rinsed in propylene oxide, and embedded overnight in 1:1 propylene oxide/Spurr’s resin and left to polymerize at 56 °C for 48 h. Ultrathin sections of 70–90 nm were cut with an ultramicrotome, EM UC7 (Leica), stained with uranyl acetate plus lead citrate, and viewed using a JEM1010 transmission electron microscope (JEOL, Japan).

### Reverse transcription and real-time PCR

Total RNAs were extracted and quantified as previously described [60]. One microgram of isolated RNA was reverse transcribed according to the manufacturer’s protocol using the miScript II RT Kit (Qiagen) for miR analysis and the SuperScript™ III Reverse Transcriptase Kit (Invitrogen, Tournai, Belgium) for mRNA. Real-time PCR was performed using 7300 Real-Time PCR System (Applied Biosystems, Halle, Belgium). Specific forward and reverse primers or references are described in Supplementary Tables S3 and S4. Relative expression levels were determined using the 2^−ΔΔCt^ method. Internal standard genes *RNU1A* and *β-actin* were used as endogenous quantity control to normalize microRNA and mRNA levels, respectively.

### Western blotting

Total cell lysates were performed using the M-PER^®^ reagent (Thermo Fisher) and nuclear/cytoplasmic proteins were extracted as previously described [60]. Buffers for proteins extraction contained anti-protease and anti-phosphatase cocktail. Protein extracts were separated by sodium dodecyl sulfate-polyacrylamide gel electrophoresis and then transferred to PVDF membrane. Unspecific binding sites were blocked by incubating membranes in phosphate-buffered saline containing 0.5% Tween-20 and 5% milk or bovine serum albumin. Immunoblots were incubated with a specific primary antibody and a horse radish peroxidase-conjugated secondary antibody. Blots were developed using an enhanced chemiluminescence method (ECL^+^/Amersham), and luminescent signal was analyzed with the ImageQuant Las 4000 Mini software (GE Healthcare). Western blot quantifications were performed using the ImageJ64 software (Supplementary Figure S11).

### Statistical analysis

Statistical analysis was performed using the Prism 7 software (GraphPad software, La Jolla, CA, USA). Data correspond to mean ± SD. Results were analyzed using paired *T* -test, one-way, two-way ANOVA (analysis of variance) followed by post hoc tests (see legends). *P* values were considered statistically significant when *p* < 0.05.

## Supplementary information

All suppl material
